# Combined Mapping of Multiple clUsteriNg ALgorithms (COMMUNAL): A Robust Method for Selection of Cluster Number, K

**DOI:** 10.1038/srep16971

**Published:** 2015-11-19

**Authors:** Timothy E. Sweeney, Albert C. Chen, Olivier Gevaert

**Affiliations:** 1 Institute for Immunity, Transplantation and Infection, Stanford University; 2 Biomedical Informatics Research, Stanford University, Stanford, CA 94305, United States.; 3 Department of Statistics, Stanford University, Stanford, CA 94305, United States.

## Abstract

In order to discover new subsets (clusters) of a data set, researchers often use algorithms that perform unsupervised clustering, namely, the algorithmic separation of a dataset into some number of distinct clusters. Deciding whether a particular separation (or number of clusters, K) is correct is a sort of ‘dark art’, with multiple techniques available for assessing the validity of unsupervised clustering algorithms. Here, we present a new technique for unsupervised clustering that uses multiple clustering algorithms, multiple validity metrics, and progressively bigger subsets of the data to produce an intuitive 3D map of cluster stability that can help determine the optimal number of clusters in a data set, a technique we call COmbined Mapping of Multiple clUsteriNg ALgorithms (COMMUNAL). COMMUNAL locally optimizes algorithms and validity measures for the data being used. We show its application to simulated data with a known K, and then apply this technique to several well-known cancer gene expression datasets, showing that COMMUNAL provides new insights into clustering behavior and stability in all tested cases. COMMUNAL is shown to be a useful tool for determining K in complex biological datasets, and is freely available as a package for R.

Unsupervised clustering methods generally attempt to classify samples according to functions of their features (such as grouping tumor samples according to their gene expression). Such clustering can give deep insight into a dataset, often allowing for discovery of functional subclasses that were previously unknown. However, there is no ‘right’ answer for the optimal number of clusters (K) in a dataset. There can thus be significant disagreement over how many clusters truly exist, or what the ‘right’ number of clusters is; for instance, in glioblastoma multiforme gene expression data, different studies have come to different conclusions for K (from 2–4)^1^,^2^,^3^. This disagreement arises when different clustering algorithms are used to separate the data, and when different clustering validity metrics are used to judge the ‘right’ number of resulting clusters. In addition, in cases where the number of parameters far outnumbers the number of measurements (i.e. p ≫ n, such as is the case for gene expression microarray data) there is often a need to subset the data for computational efficiency, and different subsets of the data can yield different results.

Cluster validity metrics are functions that help a user answer the question of whether a particular clustering of the data is better than an alternative clustering. For unsupervised clustering, where partitions are made without reference to external classes, these cluster validity metrics must rely only on internal measures of the data. Several such validity metrics exist, such as within-cluster distances (should be low) and between-cluster distances (should be high). Another example, the silhouette width, is an average measure of the difference in similarity of a sample to its assigned cluster and to the samples of the nearest neighboring cluster^4^. There is no clear answer as to which validity metric is ‘best’, however, and different validity metrics may yield different conclusions for which clustering is optimal even for the same data.

While unsupervised clustering using a single subset with a single algorithm with a single validity metric can still yield valuable insights, such methods are beginning to be replaced by integrative clustering methods that attempt to find a more stable, robust solution. Consensus clustering, for instance, takes multiple subsets of a dataset and uses repeated predictions of cluster assignment to gauge stability^5^. Another method, HOPACH, recursively partitions a dataset while seeking to optimize some clustering measure^6^. Both of these methods improve on previous single methods by repeatedly examining sub-clusters of a dataset; however, they ultimately utilize a single clustering algorithm and a single validity metric.

We hypothesize that combining information from different clustering algorithms and metrics could improve performance in determining the optimal K, since agreements between different algorithms might improve the signal-to-noise ratio across different K. We further hypothesized that integrating information from progressively larger subsets of a dataset might show a ‘map’ to optimality that is not otherwise present with a single subset. We here present our method, COmbined Mapping of Multiple clUsteriNg ALgorithms (COMMUNAL), which evaluates and combines multiple clustering algorithms and validity measures over a progressively increasing subset of variables, and then locally optimizes and integrates these methods to produce a 3D map of clustering optimality.

## Methods

### COMMUNAL Algorithm

We first identified a wide range of clustering algorithms and clustering metrics and selected those available in R packages. Only algorithms that require a user-defined input for K were chosen. The ‘cluster’^7^ and ‘clValid’^8^ packages are widely used and contain several well-implemented clustering algorithms chosen for further study (hierarchical, divisive hierarchical, agglomerative nesting, k-means, self-organizing maps^9^, partitioning around medioids, clustering for large applications^10^, and the self-organizing tree algorithm^11^). Algorithms which do not return an exact requested K (i.e., fuzzy algorithms) were left out. To evaluate multiple clustering validity statistics, we used the ‘fpc’ package^12^ cluster.stats function, which evaluates several cluster validity measures. These include the average distance between clusters, average distance within clusters, average silhouette width^4^, Calinski & Harabasz index^13^, connectivity^14^, Dunn index^15^, Dunn index 2^15^, entropy^16^, G2 coefficient^17^, G3 coefficient^18^, maximum cluster diameter, minimum cluster separation, Pearson’s gamma^15^, separation index^19^, widest gap, within cluster sum of squares, and within:between cluster distance ratio. We also implemented the gap statistic, as this is a commonly used validity measure^20^.

For all runs of COMMUNAL, we used Euclidean distance with a ward agglomeration method, and a range of K from 2:10. All of these parameters are modifiable by the end user in the R package.

### Clustering Algorithm and Validity Metric Optimization

For a given run of COMMUNAL, a dataset is split into progressively increasing subsets of variables based on descending order of variance, and each of these is clustered separately, allowing for an estimate of the stability of a given clustering over the range of variables available in the data. The number of subsets and range of variables are chosen by the user, and are by default ranked in descending order of variance across samples (thus the highest-variance set of variables is present in every tested data subset). For each subset, all clustering algorithms are run across the entire range of K. All clustering results are then assessed with all validity metrics.

Since different clustering algorithms and validity metrics may perform poorly in different datasets, the COMMUNAL algorithm tries to identify these and exclude them from further analysis. To identify clustering algorithms that are locally suboptimal, we calculate the percentage of clusters smaller than some minimum cluster size (we chose n = 3) across all runs of the algorithm. The algorithms with a percentage greater than the mean are removed.

Similarly, some validity metrics might be more accurate than others. We show that the monotonicity of a given validity measure is a reasonable proxy for accuracy (see results). The assumption is that validity measures that repeatedly return a monotonic ranking have a bias for lower or higher K, and thus less accurate in identifying an optimal K. COMMUNAL thus assesses the percentage of times a validity may be measure returned a monotonic ranking across all data subsets for all clustering algorithms. Validity measures above the median count of monotonic results are removed.

Finally, the remaining validity metrics are subjected to a correlation analysis, and are clustered based on their mutual correlation. The set of validity metrics with the lowest mean correlation within each cluster was then taken as the ‘final’ set of validity metrics. This final set of validity metrics is thus locally optimized to present stable, accurate validity information for the given dataset and algorithms under study.

### 3D Plots

The final optimized set of clustering algorithms and validity metrics are then used to assess cluster optimality across the range of K and across the increasing variable subsets. Since each clustering algorithm is tested over a range of K and a range of variable subsets (n) for each datasets, the output for a validity measure for a given algorithm ‘j’ is a K x n matrix. For all j, the matrix is centered and scaled, and then the mean of the matrices across the j algorithms is taken as the output for a single validity metric, yielding a K x n matrix of normalized validity measures (Z-measures). In the COMMUNAL R package, the K x n Z-measure matrix is plotted as a 3D topographic map using the R package ‘rgl’ (Supplemental Figure S1)^21^. The steepest non-edge local maxima are marked as potentially optimal K (red dot). This is calculated by taking the difference-of-differences for validity measures at any given variable subset, and thus roughly approximates the lowest second derivative. It cannot be calculated at the edge, so the red dots are always non-edge peaks, unless there are no internal peaks, in which case it reverts to the highest edge. The absolute maximum K for any subset is marked with a blue dot. Cluster stability is assessed by the user, as it depends heavily on the number of subsets included, which is chosen by the user.

### Core Cluster Assignments

Once the user has chosen a given optimal K at a given set of variables for a dataset, it is necessary to determine sample cluster assignments. However, arriving at a single final sample cluster assignment is not straightforward since COMMUNAL returns clustering output for several algorithms which (1) may give different arbitrary assignment names to the same clusters and (2) may disagree on sample cluster assignment. A given sample thus has a vector of cluster assignments, one from each included algorithm. We thus perform a ‘core’ clustering algorithm in two steps. First, the samples-by-algorithms matrix of cluster assignments is searched for blocks of samples with identical cluster assignment vectors. There must be at least k-1 such blocks, or the core clustering fails and samples cannot be assigned, requiring the user to select fewer algorithms over which to integrate the signal (it is always possible to use output from a single algorithm). If there are at least k-1 such blocks of samples (without which the system is under-determined), they are re-keyed to match a given algorithm’s assignment, and the resulting changes are used to re-key each remaining algorithm. This yields a new matrix where all algorithms use the same key, but may not agree on cluster assignment. If this is the case, the user may choose whether to include more algorithms or more samples (i.e., requiring only 50% overlap of all included algorithms may give results for all samples, but requiring 80% overlap of all algorithms may mean 5% of all samples are returned with a ‘0’, meaning no cluster assignment). For the results reported here, an arbitrary threshold of 50% algorithm overlap was used.

### Simulated Data

We first ran the COMMUNAL algorithm on simulated Gaussian distributed clusters generated with the ‘MixSim’ package^22^. We used three different values for K (3, 5, or 7), with 1000 informative variables and with or without 1000 noisy variables. This yielded a total of 6 test cases with a known K that was progressively more difficult to identify. The 6 simulated data cases were each split into progressively growing subsets of 200 variables ranked by decreasing variance (such that the first subset has the 200 variables with the greatest variance across samples, then the next subset has the top 400 variables with the greatest variance, etc.).

### Gene Expression Data

We downloaded normalized log2-transformed microarray gene expression data from the Cancer Genome Atlas (TCGA) from the Broad Institute GDAC Firehose (version 2014_10_17, doi:10.7908/C1K64H78) for all cancers for which more than 50 mRNA microarray samples were available (n = 7). The joint COADREAD was used, instead of the separate COAD and READ datasets. The microarray data were used as is, except that samples not part of the available Broad Institute clustering runs were also not included here, to allow for exact comparison between methods. Finally, the merged Golub leukemia dataset^23^ (downloaded from the ‘golubEsets’ R package) was also used as a separate gene expression test clustering case.

We compared COMMUNAL output for each of the TCGA datasets to Broad Institute GDAC clustering (version 2014_10_17) for all 7 TCGA cancer types. The Broad Institute analysis consists of consensus hierarchical and non-negative matrix factorization (NMF)^24^ clustering using the 1500 most variable genes in a dataset. The COMMUNAL clustering results at 1500 genes were thus specifically compared to the TCGA hierarchical cluster assignments. Contingency matrices of cluster assignments were assessed with Goodman and Kruskal’s lambda, where the fewer number of clusters was always the dependent variable (i.e., assessing whether the greater number of clusters explained the fewer number of clusters).

All analyses were performed in R. COMMUNAL is available on CRAN as an R package.

## Results

**Figure 1:**
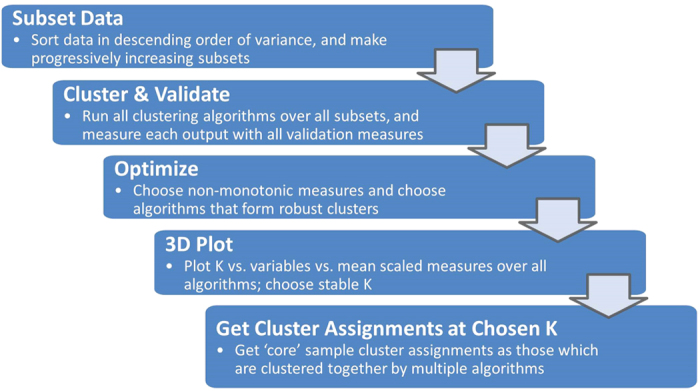
Schematic of COMMUNAL workflow.

COMMUNAL is a method for integrating results from multiple locally optimized clustering algorithms and validity measures to determine optimal stable clustering. A schematic of the COMMUNAL workflow is shown in Fig. 1, and a sample 3D COMMUNAL map is shown in Supplemental Figure S1.

### COMMUNAL in simulated data

We first ran COMMUNAL on six simulated datasets (300 samples × 1000 variables) with a known K of 3, 5, or 7, with or without 1000 added noisy variables. Each of these was progressively subsetted as described, yielding six sets of 5 or 10 subsets each. All algorithms were run over the range of K from 2–10 for all subsets. Since the simulated data has a ‘correct’ value for K, each validation measure was tested for accuracy (the presence of a peak at the correct K) for all simulated data subsets. We also tested each validity measure for monotonicity. The mean percentage of accuracy and monotonicity for each measure across all 6 simulated datasets is shown in Table 1. Notably, some metrics performed particularly poorly, nearly always ranking cluster outputs in monotonic order (e.g., cluster entropy and within-cluster sum of squares). The gap statistic was above the mean for monotonic rankings for simulated data (53%), even though it was highly accurate (25%). Notably, there was a high negative correlation between percent monotonicity and percent accuracy (Pearson r = −0.68). We thus used the monotonicity of a given measure across all subsets of a given dataset as a measure of local accuracy. The accuracy measure above was thus not used directly in selecting validity measures for the simulated data, as this would be an unfair advantage not applicable to real-world applications.

**Figure 2:**
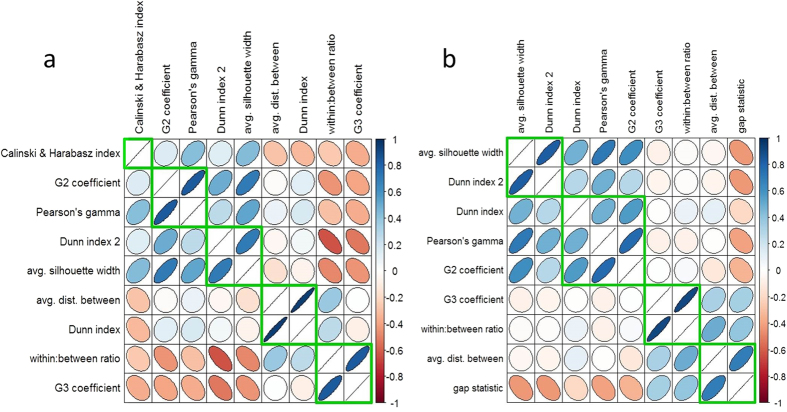
Correlations of non-monotonic validity measures across all test data sets. (**a**) Simulated data; (**b**) Broad Institute TCGA gene expression data. Green outlines show the main clusters of validity measures, which were used to inform the local choice for number of measures to include in each data type.

COMMUNAL performs a clustering analysis on the correlations of non-monotonic measures, but this requires a user-defined number of clusters. We thus examined the correlation of all measures across all simulated data to get an estimate for what a good choice for cluster number would be, and chose 5 measures as a good estimate for the simulated data (Fig. 2a).

COMMUNAL calculates the percentage of all runs (across all K and all data subsets) for which the clustering algorithms produced any clusters with fewer than 3 members (Table 2). While tiny clusters can be informative about outliers, including these can throw off the count of K (for instance, algorithm A produces two clusters of 100 and 200 samples, while algorithm B produces two clusters of 2 and 298 samples, but then splits into 2, 98, and 200 samples at K = 3; this major split in the data is thus apparent at two different K in the two different algorithms). Each COMMUNAL run thus uses only those clustering algorithms with a percentage of tiny clusters lower than the local mean. In practice, this step improved the accuracy of COMMUNAL in simulated data.

**Figure 3:**
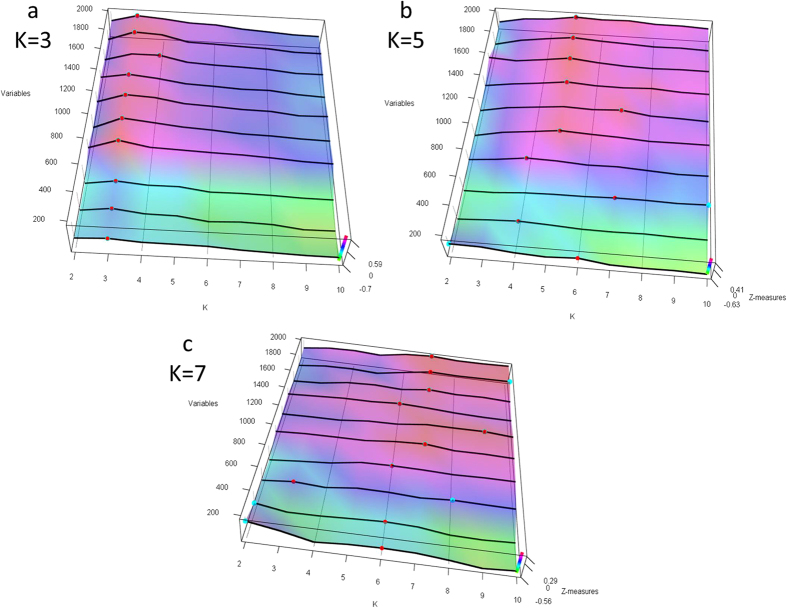
COMMUNAL output for simulated data with 1000 informative variables and 1000 noisy variables. Shown is a 3D map of the range of K vs. increasing data subsets vs. mean standardized validity measures. (**a**) Simulated K = 3. (**b**) Simulated K = 5. (**c**) Simulated K = 7.

COMMUNAL was thus run on all six simulated datasets. Even in the presence of noisy variables, the COMMUNAL algorithm was able to correctly identify the correct K in all cases (Fig. 3). Across all simulated data subsets, COMMUNAL identified the right peak more than 60% of the time, which is a substantial improvement over any lone validation measure (highest single accuracy is the Dunn index, 36%, Table 1). As expected, the case of K = 7 was the hardest to identify, with only 40% of cases correct, and the flattest peaks at the correct K.

### COMMUNAL in Broad Institute TCGA data

This COMMUNAL clustering was next performed for all cancers with microarray-based mRNA measurements in TCGA (breast cancer (BRCA)^25^, colorectal adenocarcinoma (COADREAD)^26^, glioblastoma multiforme (GBM)^1^,^27^, renal cell carcinoma (KIRC)^28^, lung squamous cell carcinoma (LUSC)^29^, ovarian cancer (OV)^30^, and uterine cancer (UCEC)). The official published TCGA clusterings for these cancers used different criteria for variable selection, different algorithms, and different validity metrics. Rather than try to mimic these various criteria, we used the standardized analysis pipeline from the Broad Institute GDAC Firehose. The Broad analyses are performed in a standard manner: for the mRNA expression data, consensus clustering (using NMF or hierarchical clustering) is performed on the 1500 most variable genes. The optimal number of clusters found by COMMUNAL (as evaluated by the authors) and the Broad Institute optimal cluster numbers for all 7 cancers are shown in Table 3, and validity measure monotonicity is shown in Table 1. COMMUNAL output for GBM and OV revealed agreement in stable K (4 and 3, respectively) with the Broad analysis at 1500 genes. However, since the Broad consensus clustering is only assessed at a single subset of 1500 genes, it is unknown whether these are robust to variable selection. In contrast, the COMMUNAL 3D normalized optimality maps showed several interesting trends over a large range of included genes (100–7500).

Since COMMUNAL selects clustering algorithms and validity measures locally for each dataset, a table of the various included metrics is shown in Supplemental Table S1. In general, there is a relatively stable group of algorithms (hierarchical, K-means, self-organizing maps, and partitioning around medioids) and metrics (gap statistic, Dunn index, Pearson’s gamma, and G3 coefficient), with 1–2 additions or subtractions for each dataset. This is not surprising given the similarity of the data. However, there was a high correlation between validity measure monotonicity in the simulated data and the gene expression data (Pearson r = 0.89, Table 1). This suggests either similarity of the simulated data to the gene expression data, invariance of the measures to the underlying data, or both. Correlation analysis of non-monotonic validity measures across all Broad Institute TCGA data revealed four clusters of measures (Fig. 2b), and so four measures were obtained for each run of COMMUNAL in gene expression data.

**Figure 4:**
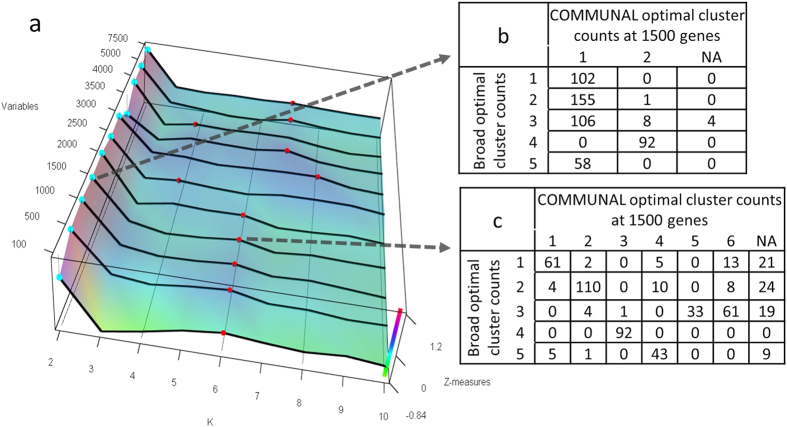
COMMUNAL output for the breast cancer (BRCA) dataset. (**a**) 3D map of K vs. genes included vs. standardized validity measures. (**b**,**c**) Comparison of COMMUNAL core cluster assignment counts vs. Broad Institute consensus-hierarchical cluster assignment counts at 1500 genes for (**b**) K = 2 (lambda = 0.87) and (**c**) K = 6 (lambda = 0.72). Broad cluster #4 is the ‘Basal’ group.

The COMMUNAL mapping of breast cancer gene expression showed stable optimality at K = 2, with a sub-optimal second peak most frequently around K = 6, then growing to K = 7 as more variables are included (Fig. 4). At K = 2, COMMUNAL divides the samples into the ‘basal’ group (COMMUNAL cluster #2) versus all other groups, reflecting that this split may be the strongest division in the gene expression data (Fig. 4b). The further clustering at K = 6 largely matches the TCGA consensus hierarchical clustering at 1500 genes (lambda = 0.72). The extra cluster appears to be a split of Broad cluster #3 into two COMMUNAL clusters (#5 and #6).

**Figure 5:**
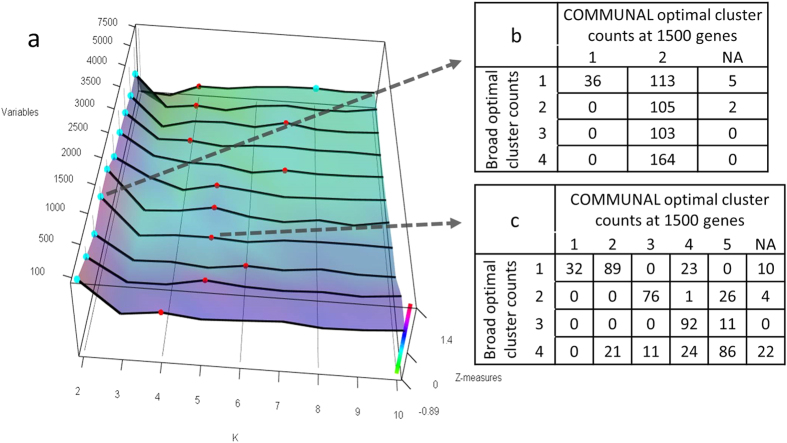
COMMUNAL output for the glioblastoma multiforme (GBM) dataset. (**a**) 3D map of K vs. genes included vs. standardized validity measures. Stable optima are seen at K = 2, and then K = 4/5. (**b**,**c**) Comparison of COMMUNAL core cluster assignment counts vs. Broad Institute consensus-hierarchical cluster assignment counts at 1500 genes for (**b**) K = 2 (lambda = 0.11) and (**c**) K = 5 (lambda = 0.64).

Glioblastoma multiforme TCGA data was previously found to have an optimal K = 2–4 in multiple analyses^1^,^2^,^3^. The COMMUNAL output shows strong optima at K = 2, as well as sub-optima at K = 4/5 (Fig. 5). At 1500 genes, the COMMUNAL K = 5 and Broad clusterings show some agreement (Fig. 5c, lambda 0.64). More importantly, the changing stability over multiple input variables suggests that prior disagreement could easily have resulted simply from choosing different subsets of the data.

The rest of the pan-cancers each had interesting findings. Colorectal adenocarcinoma (COADREAD) shows a persistent main optima at K = 2, but no sub-optima; the cluster assignments for K = 2 are strongly explained by the Broad Institute clusters (lambda = 0.83; Supplemental Figure S2).

Renal cell carcinoma (KIRC) shows very high stability, with a clear optimal K = 2, with sub-optima K = 4 (Supplemental Figure S3). This finding suggests that the clustering information present in the top 500 most variable genes in the KIRC dataset is not affected by any additional genes. Notably, the COMMUNAL KIRC clusters at 1500 genes are nearly perfectly explained by the TCGA clustering (K = 2, lambda = 1.0; K = 4, lambda = 0.97).

In contrast to KIRC, lung squamous cell carcinoma (LUSC) COMMUNAL output is notable for its change in clustering stability over inclusion of variables, with early stability at K = 2/5 giving way to K = 6 at 3000 genes (Supplemental Figure S4). The COMMUNAL K = 5 clusters at 1500 genes are largely sub-clusters of the Broad consensus clusters at K = 3 (lambda = 0.73).

Ovarian cancer (OV) shows generally unstable clustering, with five peaks at K = 3, and four at K = 6 (Supplemental Figure S5). The relatively flat map suggests that the present clusters may not be highly stable, although the steep increase at 5000 and then 7500 variables for K = 3 suggests that stability may increase for a larger gene set.

Finally, uterine cancer (UCEC) COMMUNAL output show a main peak at K = 2 and a sub-optimal peak at K = 6 (Supplemental Figure S6). There is moderate agreement at 1500 genes between the COMMUNAL clusters at K = 6 and the Broad consensus clusters at K = 5 (lambda = 0.63).

Overall, the COMMUNAL 3D maps of cluster validity provide new insights into almost every dataset examined, including gene range estimates of stability, comparative estimations of cluster validity, and qualitative assessments of robustness.

### Validation in Golub leukemia data

We tested the merged (training and testing) Golub leukemia dataset with COMMUNAL (Supplemental Figure S7)^23^. Since the Golub data included 2 different known classes of leukemia, we tested a range of K only from 2:6 instead of 2:10. COMMUNAL shows the expected optimality at K = 2 with the most variable 100 genes, but this split is explained poorly by cancer type (lambda = 0.15). Further investigation of more variables indicates a highly stable sub-optima at K = 5; here we find that an interaction between cancer type and hospital site of enrollment explains the large number of clusters (lambda = 0.58).

## Discussion

Determining the ‘right’ number of clusters during an unsupervised clustering exercise is a challenging task. Typically, a single subset of the data clustered according to a single clustering algorithm, and this is measured by a single cluster validity measure. We hypothesized that integrating information from multiple clustering algorithms and multiple validity measures would improve the signal:noise ratio and assist in identifying stable clusters. We further hypothesized that examining the effects of adding more variables to a dataset would assist in determining stability. We show here that COMMUNAL achieves both of these goals, while further optimizing the algorithms and metrics for each tested dataset to improve accuracy.

COMMUNAL was first tested with simulated data, and showed that the overall COMMUNAL optimal stable clusters far outperformed the accuracy rate of any individual validation metric across all data subsets and all clustering algorithms. We also showed that our method of using monotonicity as a metric for the optimality of a given validity measure is a reasonable proxy for accuracy.

We next applied COMMUNAL to TCGA gene expression microarray data, and compared our clustering to Broad Institute standard cluster analysis. Here we found several interesting insights. One particularly helpful use of COMMUNAL clustering is the ability to gauge the amount of instability in the final decision of optimal K. If one is to ultimately select some number of genes at some K as ‘optimal’, it is useful to know whether this is a minor local optimum or truly represents a stable optimum. So, for instance, the breast cancer data show a clear optimum at K = 2 over a large range of data (reflecting the ‘basal’ subgroup), indicating that this may the most important split in the gene expression data. Alternatively, the glioblastoma data suggest that, as the number of genes included increases, there may be some instability over whether K = 4 or K = 5 is a good sub-optimum. Finally, in the Golub leukemia dataset, COMMUNAL was able to identify batch effects that would be important to remove in any further analysis of the data. One major advantage of COMMUNAL is thus the ability to help identify both major and minor optimal clusters at a given subset, and how these change with different subsets.

Overall, interpreting the COMMUNAL output requires human intuition. First, its input also requires human intuition, especially in the number and range of variable subsets for a given dataset. We used 10–11 variable subsets for our data, but this may not be ideal. Too few subsets will interfere with the ability to make local optimizations in algorithms and metrics; too many subsets will increase computational time and may offer too many points for easy interpretation. In addition, the chosen range of K requires some intuition of the number of clusters expected. While it is possible to choose up to 2:N clusters, variations in the higher-K range may move the automatically generated optima (red/blue points) significantly, even though the lower-K range maintains its same shape. Thus, interpretability relies on appropriate input parameterization. A final issue is the question of choosing the ‘best’ optima. One method is to simply take the overall maximum of validity metrics (blue point). However, as the overall maximum may come at the end of an asymptotic plateau, another method is to take a point of most improvement. The ‘red point’ method roughly approximates the ‘steepest’ peak, and thus the sharpest improvement from the surrounding choices for K. We relied on a human estimation of majority vote to identify optima, and believe that requiring human interpretation in the final step will allow more flexibility in the ultimate use of COMMUNAL by the scientific community.

The COMMUNAL R package is designed to run with the algorithms and clustering metrics described here, but these can also be modified by the user; one can thus choose to examine a single algorithm or metric, or a different method of optimizing the input. It may be that for applications outside of microarray gene expression data, different sets of algorithms and metrics will be more informative. Moreover, our analysis was not a comprehensive examination of clustering algorithms and metrics; there may be some methods not considered here that would bring more information.

Overall, the COMMUNAL method for mapping unsupervised clustering integrates information from multiple subsets, algorithms, and validity metrics (optimized to the local data) to provide the user with a 3D map of cluster optimality. This map can assist in determining a robust choice of optimal cluster number, K, and can also give some qualitative confidence about this choice. Our method correctly identifies K in ‘known’ examples and also provides new insights into gene expression microarray data.

## Additional Information

**How to cite this article**: Sweeney, T. E. *et al.* COmbined Mapping of Multiple clUsteriNg ALgorithms (COMMUNAL): A Robust Method for Selection of Cluster Number, K. *Sci. Rep.* **5**, 16971; doi: 10.1038/srep16971 (2015).

## Supplementary Material

Supplemental Figures and Tables

## Figures and Tables

**Table 1:** Performance of the tested validation measures across all simulated data and all Broad Institute TCGA data.

Validity Measures	Simulated data percent accurate		Simulated data percent monotonic		Broad Institute TCGA mRNA percent monotonic	
	mean	s.d.	mean	s.d.	mean	s.d.
avg. dist. between	22	5	14	13	25	20
avg. dist. within	5	5	60	5	52	19
avg. silhouette width	34	8	8	3	7	7
Calinski & Harabasz index	12	4	48	6	70	10
connectivity	3	1	70	2	72	5
Dunn index	36	12	36	9	13	9
Dunn index 2	31	10	8	5	18	11
entropy	0	1	82	4	92	3
G2 coefficient	15	6	32	14	7	6
G3 coefficient	19	10	23	15	7	7
gap statistic	25	25	53	25	29	20
max. diameter	28	8	69	5	72	10
min. separation	17	7	66	9	62	9
Pearson’s gamma	32	9	3	4	6	5
separation index	18	12	50	1	57	10
widest gap	27	15	54	6	56	9
within cluster sum of squares	0	1	99	1	94	5
within:between ratio	11	5	35	12	8	6

Median monotonic percentage for simulated data, 49.1%; for Broad Institute data, 40.1%.

Shown is the percent accuracy of each measure compared to the known K for all simulated data, as well as the percent of all cluster runs ranked monotonically by each measure. s.d., standard deviation.

**Table 2:** Performance of the clustering algorithms across all simulated data and all Broad Institute TCGA data.

	Hierarchical	K-means	Divisive hierarchical	Self-organizing maps	Self-organizing trees	Partitioning around medioids	Clustering for large applications (CLARA)	Agglomerative Nesting
A. Simulated Data								
K3 No Noise	0	0	1.9	5.6	0	0	13.7	0
K3 Noise 1000	0	0	5.7	5.6	0	0	6.1	0
K5 No Noise	0	0	7.4	5.6	0	0	16.3	0
K5 Noise 1000	0	0	5.2	5.6	0	0	14.6	0
K7 No Noise	0	0	9.3	5.6	0	0	14.8	0
K7 Noise 1000	0	0	0.6	5.6	0	0	20	0
B. Broad Institute TCGA Data								
BRCA	0	0	20.2	1	2.4	0	12.5	0
COADREAD	0	0	37	2.2	12.3	1.3	19.4	0
GBM	0	0	15	0	18.7	0	1.3	0
KIRC	7.1	6.9	35.9	6.9	22.2	15.8	20.7	10.4
LUSC	0.3	0.3	20.2	1.3	10.4	1.3	16.2	1.5
OV	0	0	22.6	1.2	0.2	0	11.6	0
UCEC	4.9	2.5	32.5	5.1	16.3	22.7	21.7	9.3

Shown is the percent of runs for which each algorithm produced any clusters with three or fewer clusters, across all subsets.

**Table 3:** Overall optimality calls for TCGA data, including both COMMUNAL and the TCGA clustering assignments. For COMMUNAL output, if two values for K both showed repeated optima, both are listed.

	COMMUNAL major optima	COMMUNAL minor optima	COMMUNAL optima @ 1500 genes	Broad Analysis consensus NMF	Broad Analysis consensus hierarchical
BRCA	2	6 & 7	2 & 6	8	5
COADREAD	2	None	2	4	4
GBM	2	4 & 5	2 & 5	4	4
KIRC	2	4	2 & 4	3	7
LUSC	2	5 & 6	2 & 5	4	3
OV	None	3 & 6	6	3	3
UCEC	2	6	2 & 6	4	5

Note that the two TCGA consensus clustering methods (NMF and hierarchical) agree in only three cases.

For COMMUNAL output, if two values for K both showed repeated optima, both are listed
