# Supplementary material for: Combined Mapping of Multiple clUsteriNg ALgorithms (COMMUNAL): A Robust Method for Selection of Cluster Number, K
Source: Sci Rep. 2015 Nov 19;5:16971. doi: 10.1038/srep16971 (PMC4652212; doi:10.1038/srep16971)
Supplement: Supplemental Figures and Tables [file srep16971-s1.pdf]

Supplementary Information

COmbined Mapping of Multiple clUsteriNg ALgorithms (COMMUNAL): A Robust Method for Selection of Cluster Number, K

Timothy E Sweeney, MD, PhD [1,2,†,*], Albert Chen, MS [3,†], Olivier Gevaert, PhD [2,*]

1 – Institute for Immunity, Transplantation and Infection, Stanford University

2 – Biomedical Informatics Research, Stanford University

3 – Department of Statistics, Stanford University

† - These authors contributed equally to this work

* - Corresponding authors: tes17@stanford.edu, olivier.gevaert@gmail.com

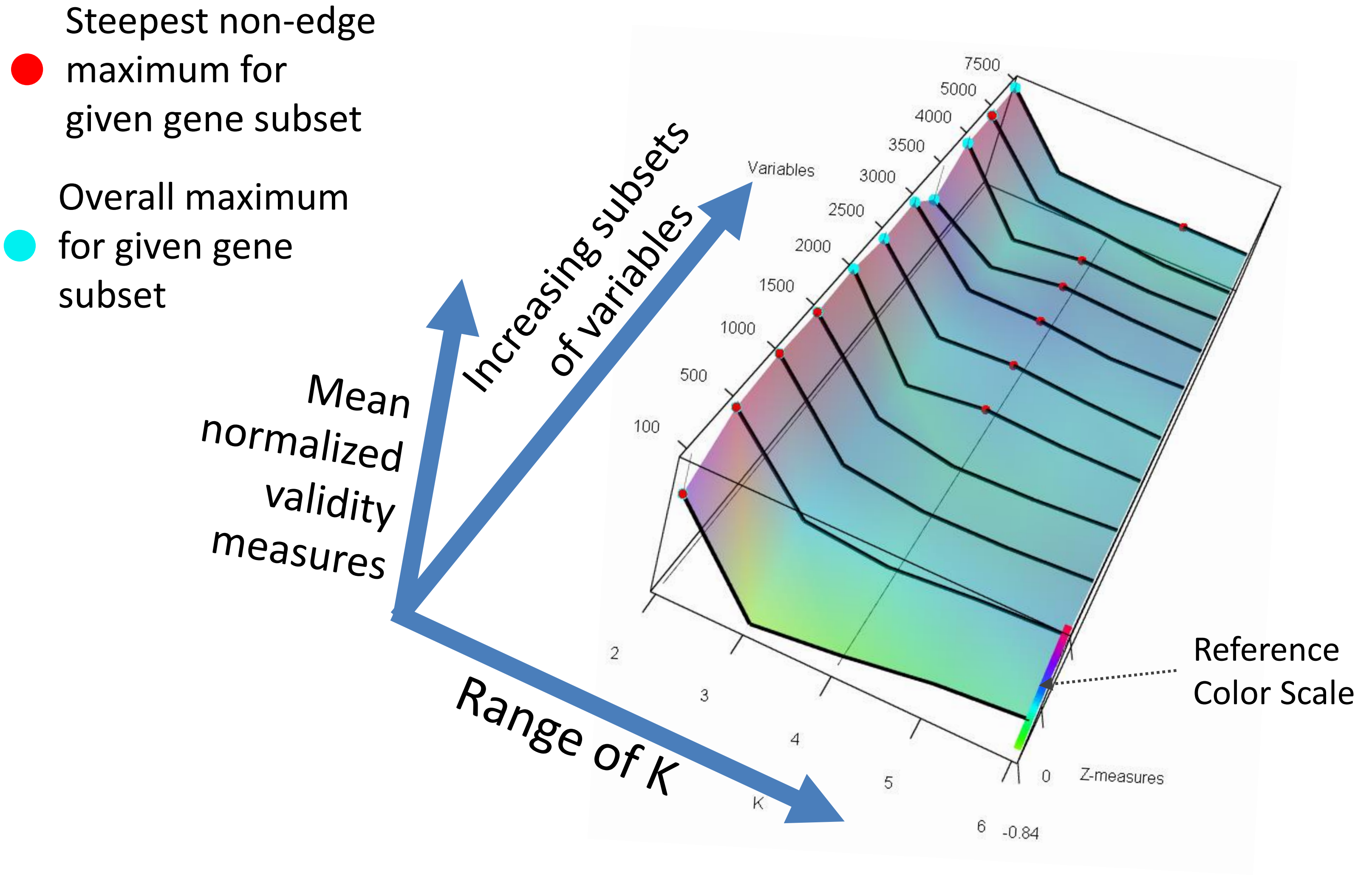

Supplemental Figure S1. Schematic of example COMMUNAL 3D map. Axis labels are shown at left. Red dots indicate steepest non-edge maximum for given gene subset; pale blue dots indicate overall maximum for given subset.

A

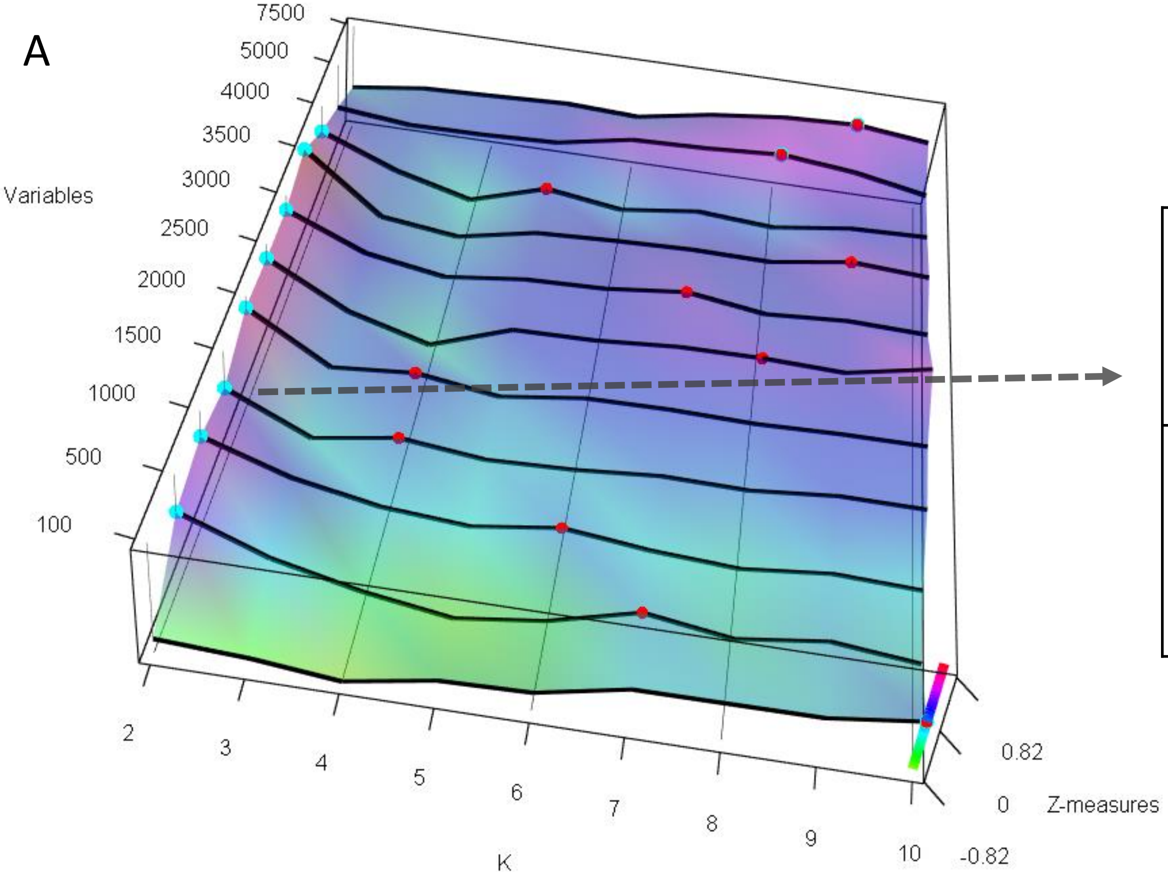

B		COMMUNAL optimal cluster counts at 1500 genes		
		1	2	NA
Broad optimal cluster counts	1	48	2	2
	2	0	41	0
	3	4	72	1
	4	0	52	0

Supplemental Figure S2. COMMUNAL output for the Broad colorectal adenocarcinoma (COADREAD). (A) 3D map of K vs. genes included vs. standardized validity measures showing optima at K=2. (B) Comparison of COMMUNAL core cluster assignment counts vs. Broad CCP-hierarchical cluster assignment counts at 1500 genes for K=2 (lambda= 0.83).

A

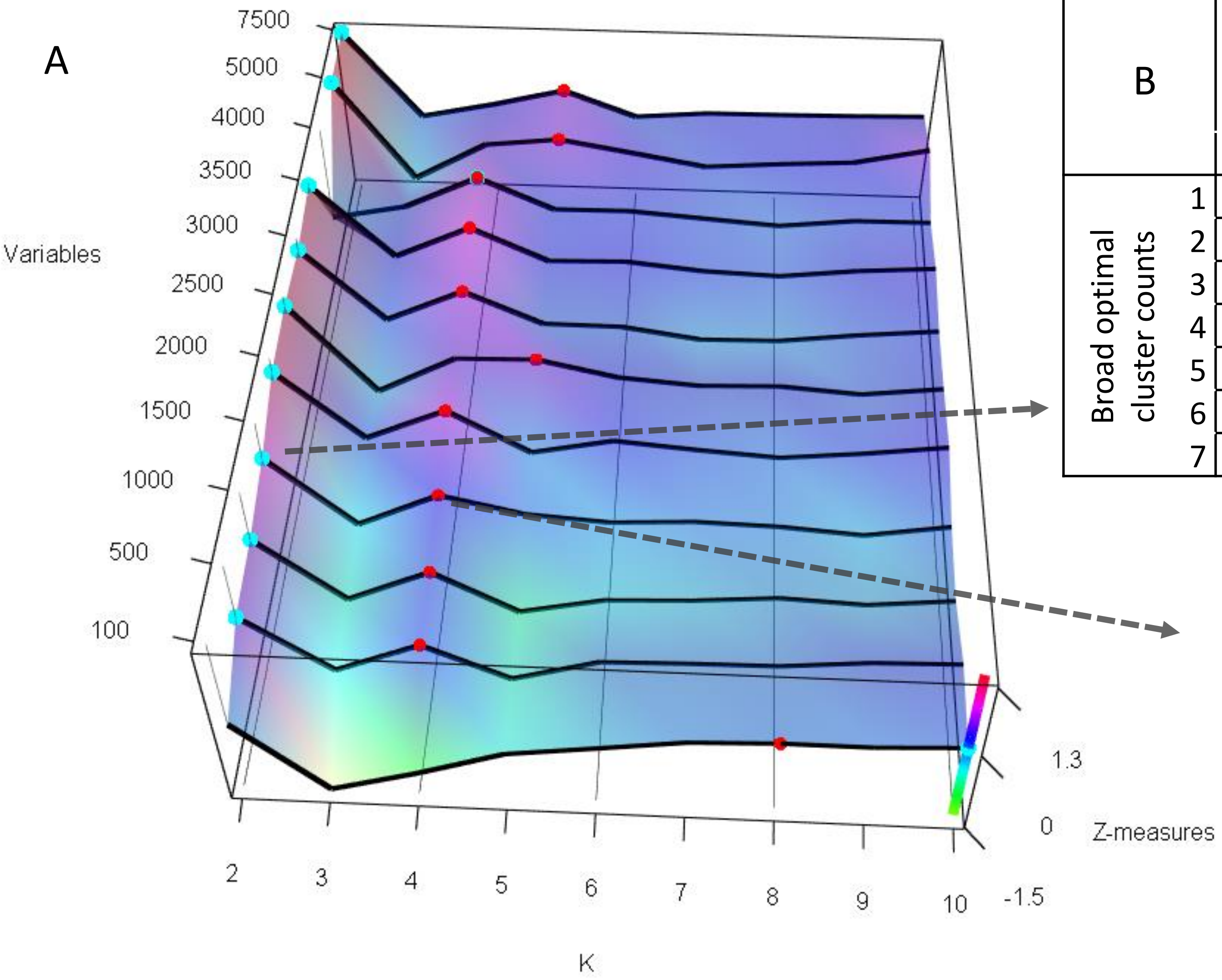

B

		COMMUNAL optimal cluster counts at 1500 genes	
		1	2
Broad optimal cluster counts	1	12	0
	2	12	0
	3	4	0
	4	11	0
	5	13	0
	6	9	0
	7	0	11

C

		COMMUNAL optimal cluster counts at 1500 genes			
		1	2	3	4
Broad optimal cluster counts	1	12	0	0	0
	2	0	12	0	0
	3	0	0	4	0
	4	0	11	0	0
	5	13	0	0	0
	6	9	0	0	0
	7	0	0	1	10

Supplemental Figure S3. COMMUNAL output for the Broad renal cell carcinoma (KIRC). (A) 3D map of K vs. genes included vs. standardized validity measures showing highly stable optima at K=2 and K=4. (B, C) Comparison of COMMUNAL core cluster assignment counts vs. Broad CCP-hierarchical cluster assignment counts at 1500 genes for (B) K=2 (lambda= 1.0) and (C) K=4 (lambda=0.97).

A

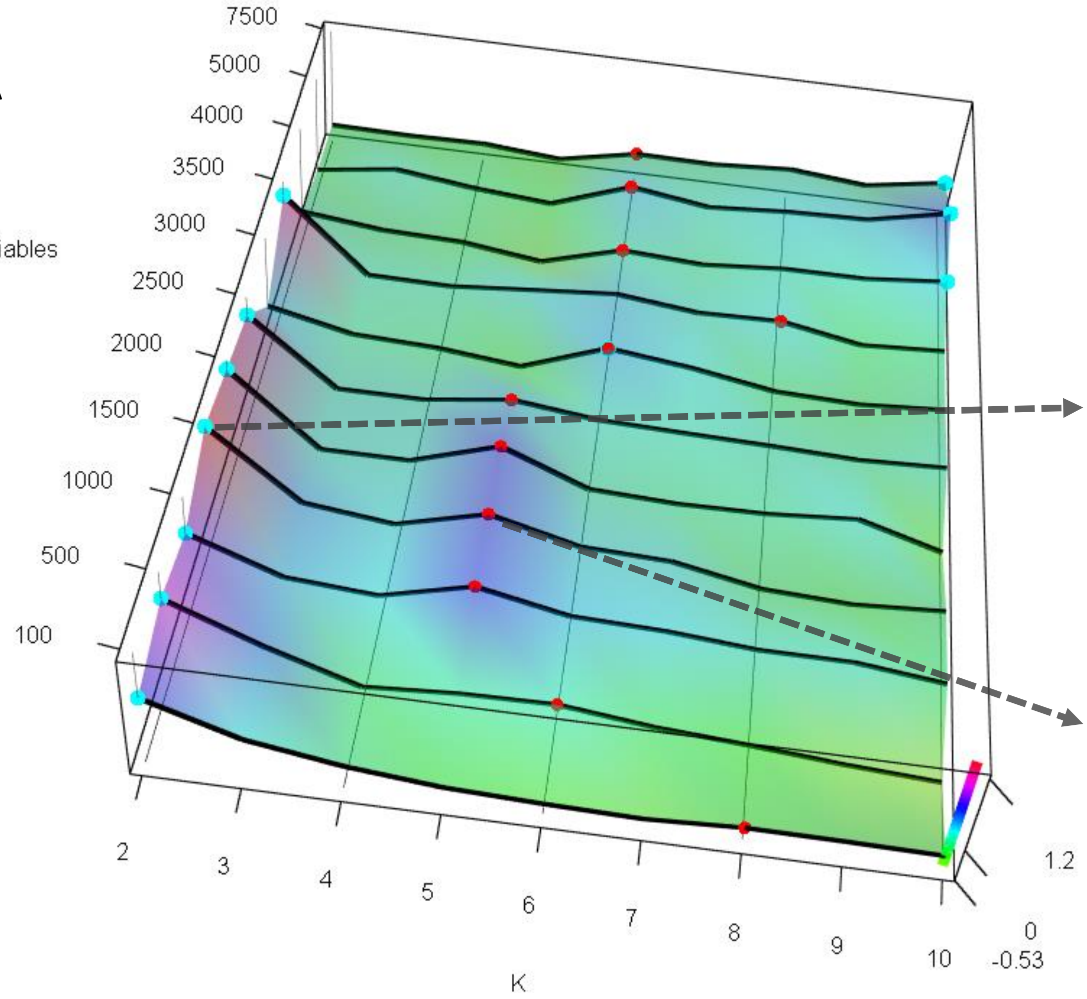

B		COMMUNAL optimal cluster counts at 1500 genes	
		1	2
Broad optimal cluster counts	1	46	1
	2	56	0
	3	24	27

C		COMMUNAL optimal cluster counts at 1500 genes					
		1	2	3	4	5	NA
Broad optimal cluster counts	1	14	0	16	0	0	17
	2	0	53	1	0	0	2
	3	0	0	22	13	9	7

Supplemental Figure S4. COMMUNAL output for lung squamous cell carcinoma (LUSC). (A) 3D map of K vs. genes included vs. standardized validity measures showing stability, at K=2 and then K=5→6. (B, C) Comparison of COMMUNAL core cluster assignment counts vs. Broad CCP-hierarchical cluster assignment counts at 1500 genes; (B) at K=2, lambda=0.27; (C) at K=5, lambda=0.73.

A

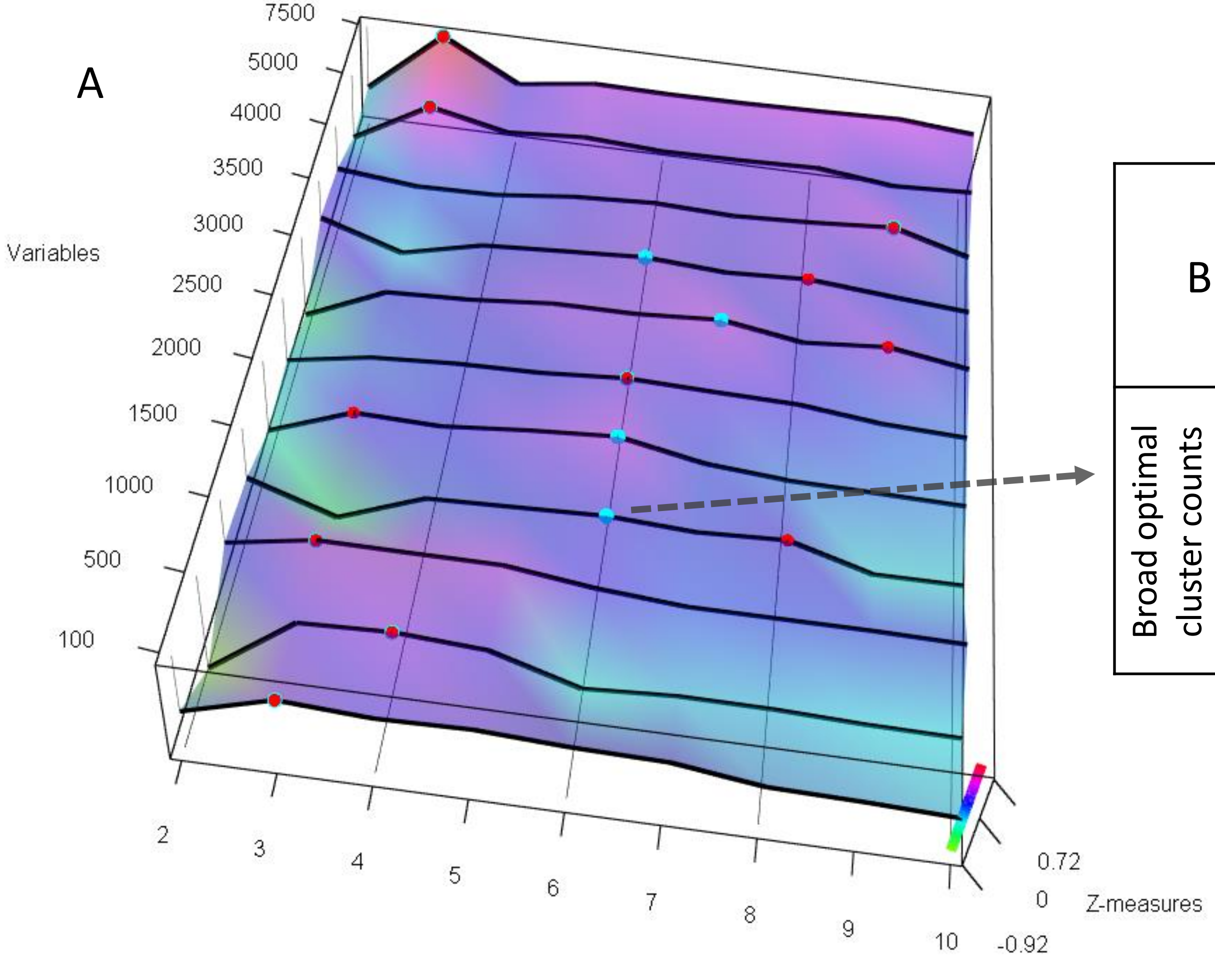

B		COMMUNAL optimal cluster counts at 1500 genes						
		1	2	3	4	5	6	NA
Broad optimal cluster counts	1	106	19	0	10	69	17	96
	2	3	141	0	0	8	0	10
	3	3	5	0	75	0	0	7

Supplemental Figure S5. COMMUNAL output for ovarian cancer (OV). (A) 3D map of K vs. genes included vs. standardized validity measures has no stable clustering, showing the importance of testing over multiple gene sets. (B) Comparison of COMMUNAL core cluster assignment counts vs. Broad CCP-hierarchical cluster assignment counts at 1500 genes; at K=6, lambda=0.74.

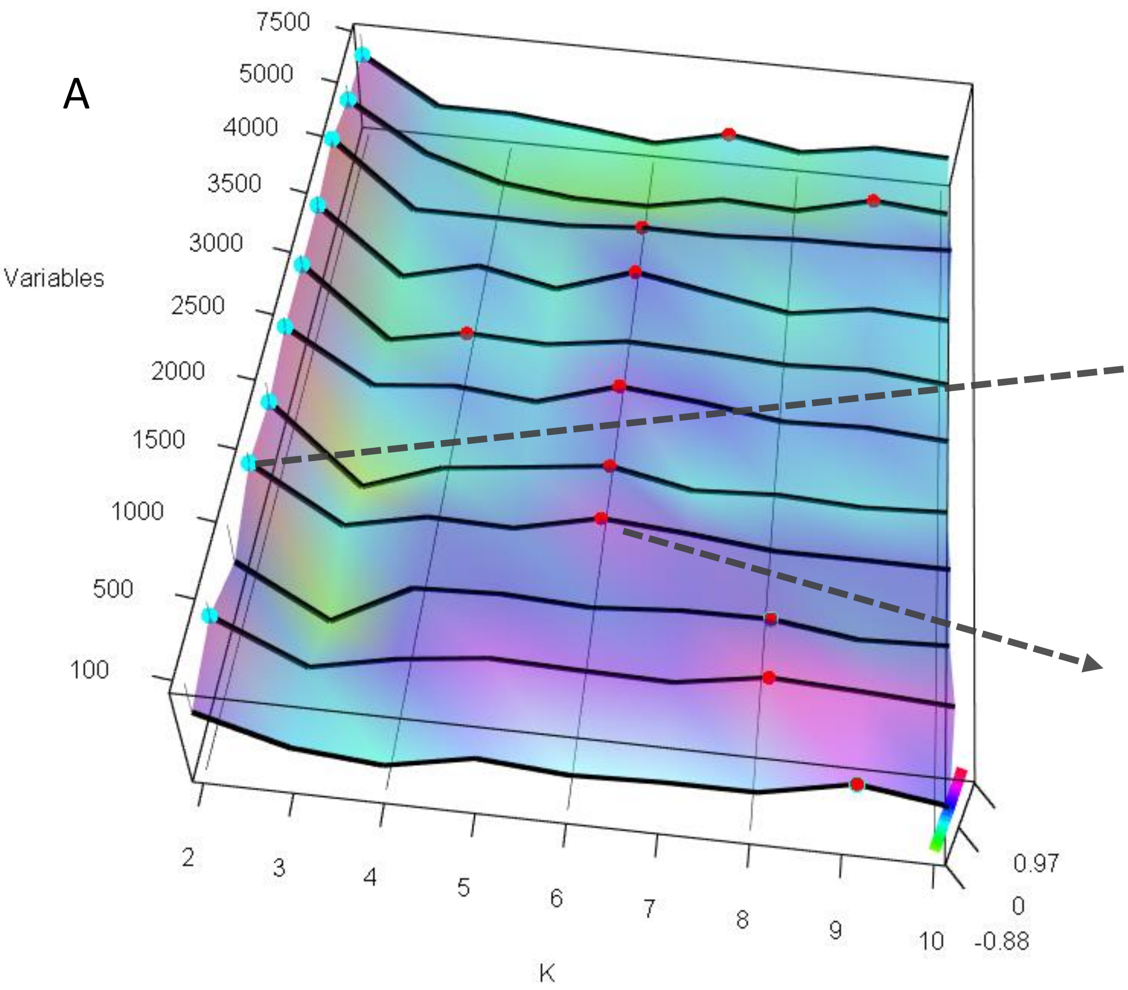

B		COMMUNAL optimal cluster	
		1	2
Broad optimal cluster counts	1	9	5
	2	10	0
	3	0	7
	4	0	7
	5	0	16

C		COMMUNAL optimal cluster counts at 1500 genes						
		1	2	3	4	5	6	NA
Broad optimal cluster counts	1	3	4	2	0	0	1	4
	2	0	0	0	0	0	10	0
	3	0	0	6	0	0	0	1
	4	0	0	7	0	0	0	0
	5	0	0	0	12	0	0	4

Supplemental Figure S6. COMMUNAL output for uterine corpus endometrial carcinoma (UCEC). (A) 3D map of K vs. genes included vs. standardized validity measures shows unstable clustering except at K=2. (B, C) Comparison of COMMUNAL core cluster assignment counts vs. Broad CCP-hierarchical cluster assignment counts at 1500 genes for (B) K=2 (lambda= 0.74) and (C) K=6 (lambda=0.63).

A

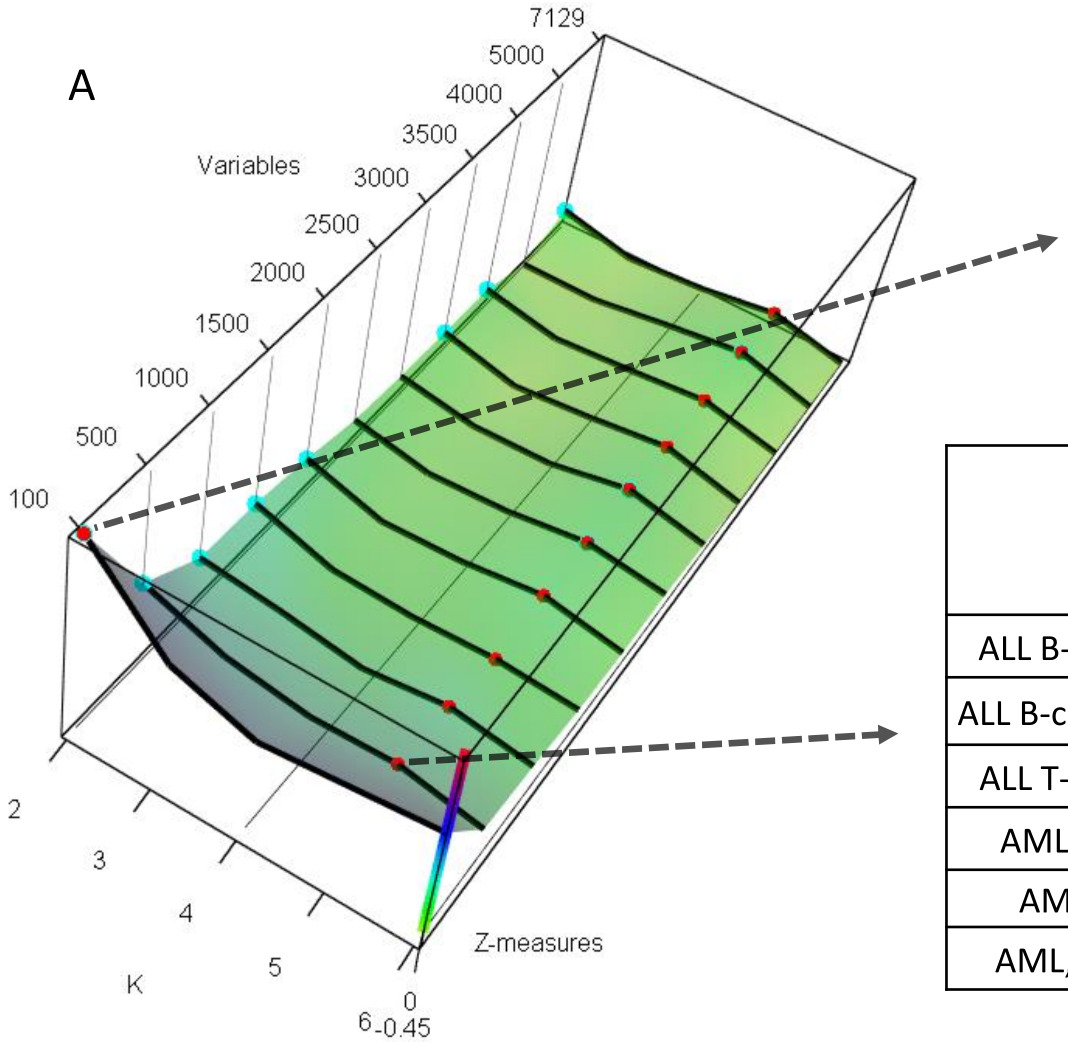

B

Type	COMMUNAL optimal cluster counts at 100 genes: 1	2	NA
ALL B-cell	34	3	1
ALL T-cell	7	2	0
AML	16	8	0

C

	COMMUNAL optimal cluster counts at 1500 genes: 1	2	3	4	5	NA
ALL B-cell, DFCI	29	2	2	0	2	0
ALL B-cell, St-Jude	0	3	0	0	0	0
ALL T-cell, DFCI	1	0	1	0	7	0
AML, CALGB	1	1	2	9	0	1
AML, CCG	1	2	1	1	0	0
AML, St-Jude	0	5	0	0	0	0

Supplemental Figure S7. COMMUNAL assessment of the entire Golub leukemia dataset. (A) 3D map of K vs. genes included vs. standardized validity measures showing optima at K=2 and K=4. (B) Core cluster assignment counts at K=2 at 100 genes vs. main leukemia type. (C) Core cluster assignment counts at K=4 at 500 genes vs. leukemia subtypes (AML, ALL-T-Cell and ALL-B-Cell); lambda = 0.15. (D) Core cluster assignment counts at K=5 at 500 genes vs. hospital enrollment sites; lambda = 0.58.

Supplemental Table S1. Listing of algorithms and metrics used in each 3D plot of COMMUNAL.

**BRCA**	
Algorithms	hierarchical, kmeans, som, sota, pam, agnes
Measures	gap statistic, dunn index 2, dunn index, g3
**COADREAD**	
Algorithms	hierarchical, kmeans, som, pam, agnes
Measures	gap statistic, dunn index, g3, dunn index 2
**GBM**	
Algorithms	hierarchical, kmeans, som, pam, clara, agnes
Measures	Widest gap, dunn index, g3, min. separation
**KIRC**	
Algorithms	hierarchical, kmeans, som, agnes
Measures	gap statistic, avg. between, g2, g3
**LUSC**	
Algorithms	hierarchical, kmeans, som, pam, agnes
Measures	gap statistic, pearson gamma, dunn index, dunn index 2
**OV**	
Algorithms	hierarchical, kmeans, som, sota, pam, agnes
Measures	gap statistic, pearson gamma, g3, dunn index 2
**UCEC**	
Algorithms	hierarchical, kmeans, som, agnes
Measures	gap statistic, g2, dunn index, dunn index 2
**Golub**	
Algorithms	hierarchical, kmeans, som, pam, clara, agnes
Measures	gap statistic, widest gap, g3, dunn index 2, avg. silhouette
